# A Brief Introduction to Management of LQTS

**DOI:** 10.31083/j.rcm2305164

**Published:** 2022-05-05

**Authors:** Richard G. Trohman

**Affiliations:** ^1^Section of Electrophysiology, Division of Cardiology, Department of Medicine, Rush University Medical Center, Chicago, IL 60612, USA

In this special issue of *Reviews in Cardiovascular Medicine* we explore “The Management of Long QT Syndromes”. In broad terms, there are two types of long QT syndromes (LQTS), congenital and acquired [1]. Torsades de Pointes (TdP), first described by Dessertenne in 1966 [2], may self-terminate or degenerate into fatal ventricular fibrillation. TdP may result from either cause of QT prolongation.

Congenital (heritable) long QT syndromes were first described in the 1950s and 1960s. Seventeen genes have been subsequently linked to LQTS [3]. The Clinical Genome Resource (ClinGen), has recent analyzed and reclassified a group of these genes to disputed or limited evidence, leaving seven genes with strong or definitive evidence of causality [3, 4]. Dual genetic abnormalities may complicate management. In genotype negative families, a more complex polygenic architecture may be responsible for QT prolongation [3, 5].

The most common subtypes of congenital LQTS are LQT1 (loss-of-function mutation in the gene [*KCNQ1*] that encodes the slow delayed rectifier current $I_{\text{Ks}}$), LQT2 (loss-of-function mutation in the gene [*KCNH2*] that encodes the rapid delayed rectifier current $I_{\text{Kr}}$) and LQT3 (gain-of-function variants in the gene [*SCN5A*], that encodes the fast inward cardiac sodium current $I_{\text{Na}}$). In general, management of congenital LQTS involves lifestyle adjustment, beta blockade, avoiding QT prolonging drugs, avoidance and/or prompt correction of electrolyte abnormalities (particularly hypokalemia), implantable cardioverter defibrillators (ICDs), and left cardiac sympathetic denervation (LCSD), particularly for individuals refractory to or intolerant of beta blockers, recipients of frequent ICD shocks, or as a bridge to ICD placement in young children [3, 6].

Benefits of cardiac pacing in congenital LQTS are less studied and less well defined than its role in acquired LQTS. TdP may be bradycardia or tachycardia dependent in congenital LQTS [7]. There may be benefit for individuals with LQT2 where arrhythmias are almost always pause dependent. LQT3 is particularly associated with QTc prolongation at slow heart rates, suggesting pacing may be beneficial.

Enhanced understanding of the late inward sodium current’s ($I_{\text{Na-L}}$) role in prolonging action potential duration and the QT interval (particularly at low heart rates) has provided evidence that additional pharmacological therapy such as mexiletine, flecainide, or ranolazine (blockers of the late inward sodium current) may be helpful for arrhythmia prevention in LQT3. Mexiletine has also been shown to decrease the corrected QT interval in LQT2 [3, 8, 9].

Acquired LQTS most frequently results from drug-induced QT prolongation. The most common drugs that that prolong QTc block the rapid component of the delayed rectifier potassium current $I_{\text{Kr}}$ via hERG channel blockade [10, 11]. In some instances, drugs that block $I_{\text{Kr}}$ also enhance $I_{\text{Na-L}}$. The most comprehensive resource for drugs to be aware of [12], categorizes them as having: (1) Known TdP Risk; (2) Possible TdP Risk; (3) Conditional TdP Risk; and (4) Drugs to Avoid in Congenital Long QT. It is available at https://crediblemeds.org/druglist [12].

Risk factors for drug-induced TdP include female gender, heart failure/cardiomyopathy, diastolic dysfunction, myocardial ischemia/infarction, left ventricular hypertrophy, valvular heart disease, treatment with multiple QT prolonging drugs or agents that interfere with their metabolism, greater than average drug dosage, baseline QTc prolongation (≥450 milliseconds), familial congenital LQTS, and prior drug-induced TdP. Liver or kidney dysfunction, bradycardia, and atrioventricular block also increase TdP risk [11, 13, 14].

Additional precipitants of acquired LQTS include electrolyte abnormalities (most commonly hypokalemia, hypomagnesemia, and less commonly hypocalcemia), hypothyroidism, and hypothermia. Cardioversion of a rapid supraventricular tachycardia (e.g., atrial fibrillation) after receiving a QT prolonging drug may result in QT prolongation. Recent evidence suggests a high prevalence of QTc interval prolongation in patients with anti-SSA/Ro antibodies, autoimmune and inflammatory diseases [14]. These factors may precipitate acquired LQTS or add to the risk of drug-induced LQTS.

All patients exposed to $I_{\text{Kr}}$-blocking agents do not develop QT prolongation. Exaggerated QT prolongation and arrhythmias are even less likely. In addition, not every patient with a *KCNH2 *loss-of-function mutation displays QT interval prolongation. While it seems that subclinical congenital long QT syndrome mutations contribute to drug-induced TdP risk, the degree to which they explain the risk remains uncertain. This apparent clinical disconnect and the increasing recognition that normal repolarization is the result of a complex interaction among multiple components, led (in the late 1990s) to the concept of “repolarization reserve”. Repolarization is achieved via multiple channels ($I_{\text{Kr}}$, $I_{\text{Ks}}$, $I_{\text{Ca-L}}$, $I_{\text{Ca-T}}$, $I_{\text{Na-L}}$, $I_{\text{NCX}}$, etc.) and repolarization reserve suggests that a reduction in $I_{\text{Kr}}$ might generate a huge effect in cells, or in patients, when other efficient repolarization mechanisms are lacking. In contrast, the same $I_{\text{Kr}}$ reduction might result in little change in repolarization time when other mechanisms can readily accomplish normal repolarization [15, 16, 17].

It is widely believed that TdP initiating beats result from early afterdepolarization (EAD)-triggered focal activity from the subendocardial Purkinje network. EADs occur during late phase 2 or phase 3 when action potential duration is increased. The mechanism(s) responsible for perpetuating TdP remain controversial [10].

The initial therapy of choice for acute drug-induced TdP is intravenous magnesium sulfate. Immediate defibrillation is indicated if sustained, hemodynamically unstable polymorphic ventricular tachycardia or ventricular fibrillation develops.

Overdrive transvenous pacing is highly effective in shortening QTc and preventing arrhythmic recurrence. It is particularly useful when TdP is refractory to magnesium or when a pause or bradycardia precipitates TdP. Short-term pacing rates of 90 to 110 beats/minute are usually employed. When temporary pacing is unavailable or during preparation for transvenous catheter placement, isoproterenol titrated to a heart rate ≥90, is useful. Isoproterenol is contraindicated in patients with congenital LQTS or ischemic heart disease [11].

It is requisite that QT prolonging medications and drugs interfering with their metabolism be promptly discontinued and avoided (if possible) in the future. Electrolyte imbalances must be corrected (serum potassium should be maintained at a level of 4.5–5 mmol/L) [12].

In the setting of acquired long QT syndrome, TdP is almost invariably preceded by a pause followed by a markedly prolonged QT interval. TdP does not appear to occur when the effective pacing rate is >70 beats per minute. However, despite programmed rates >70 beats per minute TdP may appear in the presence of pause promoting programming or oversensing [18]. In a report on congenital LQTS, most pauses that led to TdP were indisputably longer than the preceding ventricular rate. The shortest precipitating pause was 760 ms (equivalent to 79 beats/minute) and the authors recommended a minimum pacing rate of 80 beats/minute [19].

While imperfect, management of acquired LQTS is more straightforward than management of congenital LQTS. In a recent review, expert commentary on the management of 4 cases of congenital LQTS revealed agreement on 10 points, disagreement on 3, and acknowledged gaps in knowledge about 8 points [20]. While this special issue of *Reviews in Cardiovascular Medicine* is unlikely to solve all disagreements or fill every knowledge gap, we hope to enhance readers understanding of LQTS and point future investigations in a direction that will improve our therapeutic armamentarium.
